# Mediation of the association between screen time and suicidality by overweight/obesity and perceived overweight: results from the youth risk behavior surveillance system of the United States

**DOI:** 10.3389/fpsyt.2024.1287021

**Published:** 2024-03-04

**Authors:** Hong Gao, Yifei Wang, Xugang Wang, Ming Gao

**Affiliations:** ^1^ Department of Mental Health, The Second Hospital of Shanxi Medical University, Taiyuan, Shanxi, China; ^2^ Taiyuan University of Technology, Shanxi, China; ^3^ Shanxi Xinyue Psychological Counseling Research Center, Taiyuan, Shanxi, China; ^4^ Department of Physical Education, Shanxi Medical University, Taiyuan, Shanxi, China

**Keywords:** suicidality, screen time, overweight/obesity, perceived overweight, mediating effect, adolescents

## Abstract

**Aim:**

Adolescent suicide is a major public health concern, and modifiable risk factors associated with adolescent suicide remain poorly understood. This study aimed to assess the association between screen time and overweight/obesity and self-perceived overweigh and suicidality in adolescents.

**Methods:**

Adolescents from the United States Youth Risk Behavior Surveillance System (YRBSS) between 2013 and 2019 were included in this cross-sectional study. The outcome was suicidality, including considered suicide, made a suicide plan, attempted suicide, and injurious suicide attempt. Multivariable logistic regression model was used to investigate the associations between screen time, overweight/obesity, self-perceived overweight, and suicidality, and expressed as odds ratio (OR) and 95% confidence interval (CI). Mediation analysis was used to explore the role of overweight/obesity and self-perceived overweight on the association between screen time and suicidality.

**Results:**

A total of 30,731 adolescents were included, of which 6,350 (20.65%) had suicidality, including 5,361 (17.45%) with considered suicide, 4,432 (14.42%) with made a suicide plan, 2,300 (7.45%) with attempted suicide, and 677 (2.21%) with injurious suicide attempt. Adolescents with screen time ≥3h were related to higher odds of suicidality (OR=1.35, 95%CI: 1.23-1.46), overweight/obesity (OR=1.27, 95%CI: 1.19-1.38), and self-perceived overweight (OR=1.38, 95%CI: 1.30-1.48) after adjusting confounders. Adolescents with overweight/obesity (OR=1.30, 95%CI: 1.19-1.43) and self-perceived overweight (OR=1.54, 95%CI: 1.39-1.70) were associated with higher odds of suicidality. The association between screen time and suicidality was 4.67% mediated by overweight/obesity and 9.66% mediated by self-perceived overweight. Moreover, the mediating role of overweight/obesity was observed only in females, whereas there were no sex differences in the mediating effect of self-perceived overweight.

**Conclusion:**

Both overweight/obesity and self-perceived overweight mediated the association between screen time and suicidality.

## Introduction

Adolescent suicide is a major and preventable public health concern and the second most common adolescent death worldwide (1). Suicide deaths increased by 6.7% between 1990 and 2016, with more than 0.8 million deaths by suicide in 2016 (2). A meta-analysis showed that the overall prevalence of suicidal ideation among adolescents was 16.9% in low- and middle-income countries worldwide (3). Adolescent suicide is multifactorial, including age, sex, violent/bullying behavior, depressive symptom, and social isolation (4, 5). Therefore, identifying modifiable risk factors for adolescent suicide plays an important role in the development of effective adolescent suicide prevention intervention programs.

Recently, several studies have found that health-related lifestyle factors such as overweight/obesity, and physical activity (sedentary) were associated with adolescent suicidality (6–8). It was reported that overweight/obese adolescents were at higher risk for suicidal ideations and attempts than normal weight adolescents (6, 7). Adolescents who perceived themselves as overweight were also at higher risk for suicide-related behaviors (8, 9). These phenomena may be related to the fact that adolescents with overweight/obesity (or perceived-overweight) were more likely to experience depressive symptoms, self-stigma, and bullying (10–12). Moreover, excessive screen time has also been reported to be related to higher adolescent suicidality (13, 14), which may be due to poorer mental health, poorer sleep quality, and higher risk of cyber bullying among adolescents with more screen time (15, 16). Excessive screen time was also a risk factor for adolescent overweight and perceived-overweight (17, 18). Therefore, we hypothesized that overweight and perceived-overweight may mediate the association between screen time and suicidality in adolescents.

This study aimed to explore the association between screen time and overweight/obesity and self-perceived overweigh and suicidality. The mediating effect of overweight/obesity and self-perceived overweigh in the association between screen time and suicidality was investigated.

## Methods

### Design and participants

Data for this cross-sectional study were drawn from the Youth Risk Behavior Surveillance System (YRBSS) between 2013 and 2019. The YRBSS is a publicly accessible database that was developed in 1990 by the Centers for Disease Control and Prevention (CDC) to monitor health risk behaviors established in childhood and early adolescence that are associated with death, disability, and social problems among youth and adults in the United States (available at: https://www.cdc.gov/healthyyouth/data/yrbs/overview.htm). The YRBSS uses a three-stage cluster sampling design with a target population of students in grades 9 through 12, and these surveys are conducted every two years. A self-administered computer-scanned questionnaire was used for the survey that was anonymous and voluntary and had parental permission. The protocol of YRBSS were approved by the institutional review board of the CDC and is publicly available. Participants aged 14-18 years old with complete information on suicide behaviors, digital device use, self-perceived weight, and body mass index (BMI) were included. Participants with missing information on important covariates, such as physical activity, drinking alcohol, cyber bullying, and school bullying, were excluded.

### Outcomes

The outcome of this study was the suicidality of adolescents. Suicidality includes considered suicide, made a suicide plan, attempted suicide, and injurious suicide attempt. Adolescents were considered to have suicidality if they had any of these four suicidalities. Suicidality was measured through the YRBSS questionnaire (https://yrbs-explorer.services.cdc.gov/#/). The type of “considered suicide” was measured by the survey question, “During the past 12 months, did you ever seriously consider attempting suicide?”. The type of “made a suicide plan” was measured by the survey question, “During the past 12 months, did you make a plan about how you would attempt suicide?”. The type of “attempted suicide” was measured by the survey question, “During the past 12 months, how many times did you actually attempt suicide?”. The type of “injurious suicide attempt” was measured by the survey question, “If you attempted suicide during the past 12 months, did any attempt result in an injury, poisoning, or overdose that had to be treated by a doctor or nurse?”.

### Covariates

The characteristics of participants were collected including age (≤16 years and >16 years), sex (female and male), race (Hispanic/Latino and others), BMI, screen time (<3h and ≥3h), self-perceived overweight (yes and no), smoking (yes and no), drinking alcohol (yes and no), physical activity (yes and no), illegal drug (yes and no), cyber bullying (yes and no), school bullying (yes and no), school safety concern (yes and on), early sexual intercourse (yes and no), and sadness or hopeless (yes and no).

#### Independent variables (screen time)

Screen time consists of television viewing and computer/videogame use time. Television viewing was measured by the survey item, “On an average school day, how many hours do you watch TV?”. Computer/video game use was measured by the survey item, “On an average school day, how many hours do you play video or computer games or use a computer for something that is not school work?”. Response options were none, <1h, 1h, 2h, 3h, 4h, and 5 or more hours per day. The current study divided screen time into two categories: (1) ≥3h for either television viewing or computer/videogame use (screen time ≥3h); (2) <3h for both television viewing and computer/videogame use (screen time <3h). Excessive screen time was defined as ≥3h of screen time according to previous studies (19, 20).

#### Mediating factors (overweight/obesity and self-perceived overweight)

Adolescents were categorized into overweight/obesity and non-overweight/obesity based on age- and sex-specific BMI. For BMI by age and sex, adolescents were considered overweight when their BMI was at or above the 85th percentile, and obesity when their BMI was at or above the 95th percentile (21, 22).

Self-perceived weight was measured by the survey item, “How do you describe your weight”. Response options for this item were: 1 (very underweight), 2 (slightly underweight), 3 (the right weight), 4 (slightly overweight), and 5 (very overweight). Those who considered their weight as slightly or very overweight were categorized as self-perceived overweight (9).

### Statistical analysis

Weighting factors from the YRBSS database (PSU, Stratum, Weight) were applied to each student’s record to adjust for non-response and the oversampling of black and Hispanic students in the sample. The statistical description was performed based on the study outcomes of suicidality, considered suicide, made a suicide plan, attempted suicide, and injurious suicide attempt. Weighted prevalence rates of suicidality, considered suicide, made a suicide plan, attempted suicide, and injurious suicide attempt were reported. Weighted chi-squared test was used to analyze the differences in prevalence rates of suicidality, considered suicide, made a suicide plan, attempted suicide, and injurious suicide attempt by different characteristics. Multivariable logistic regression model was used to investigate the associations between screen time, overweight/obesity, self-perceived overweight, and suicidality, and the results were expressed as odds ratio (OR) and 95% confidence interval (CI). In analyzing the associations between screen time and overweight/obesity and self-perceived overweight, the logistic regression model was adjusted for sex, race, smoking, school bullying, and sadness or hopeless. In analyzing the associations of overweight/obesity, self-perceived, and screen time with suicidality, the logistic regression model was adjusted for sex, race, smoking, drinking alcohol, illegal drug, cyber bullying, school bullying, school safety concern, early sexual intercourse, and sadness or hopeless.

The mediation of the association between screen time and suicidality by overweight/obesity and self-perceived overweight was assessed using the “RMediation” and “jtools” packages in R software (23). The relationship of screen time with overweight/obesity and self-perceived overweight was represented by “Effect A”. The relationship of overweight/obesity and self-perceived overweight with suicidality was represented by “Effect B”. The indirect effect was “Effect A × Effect B”. The relationship between screen time and suicidality was represented by “Effect C” (total effect). Moreover, the relationship between screen time and suicidality was represented by “(Effect C’)” (direct effect) after adjusting for overweight/obesity or self-perceived overweight. Standardized coefficients were utilized to show the estimates for each pathway. The percentage of mediation effect was calculated as: [exp(Effect C) - exp(Effect A)] ×100/[exp(Effect C)-1].

All statistical analyses were performed by R 4.2.0 software (Institute for Statistics and Mathematics, Vienna, Austria). *P*-values (two-sided) less than 0.05 was considered statistically significant.

## Results

### Characteristics of included participants

**Figure 1:**
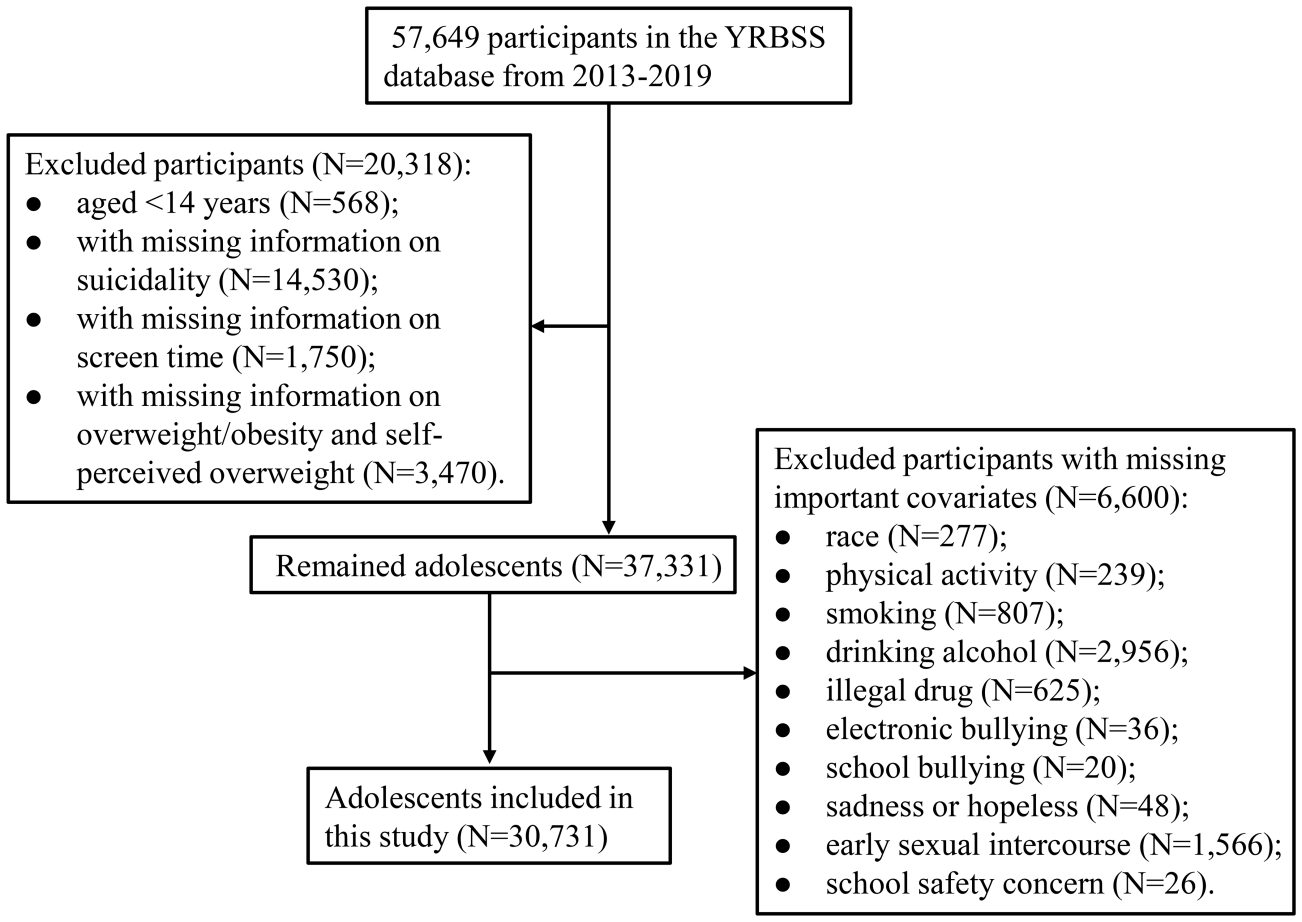
Flow chart for study population selection. YRBSS, Youth Risk Behavior Surveillance System;.

The total number of participants in the YRBSS surveys of 2013, 2015, 2017, and 2019 was 57,649. A total of 26,918 participants were excluded, including 568 participants aged <14 years, 14,530 participants with missing information on suicidality, 1,750 participants with missing information on screen time, 3,470 participants with missing information on overweight/obesity and self-perceived overweight, and 6,600 participants with missing important covariates. A total of 30,731 adolescents were included in the analysis (**Figure 1**). Of these 30,731 adolescents, 6,350 (20.65%) had suicidality, including 5,361 (17.45%) with considered suicide, 4,432 (14.42%) with made a suicide plan, 2,300 (7.45%) with attempted suicide, and 677 (2.21%) with injurious suicide attempt (**Table 1**). There were 13,579 (43.75%) adolescents with screen time ≥3h, 9,379 (38.45%) adolescents with overweight/obesity, and 10,000 (38.35%) adolescents with self-perceived overweight.

**Table 1:** Characteristics of included participants.

Variables	Overall	Suicidality	Considered suicide	Made a suicide plan	Attempted suicide	Injurious suicide attempt
	Total (n=30731)	Yes (n=6350)	Yes (n=5361)	Yes (n=4432)	Yes (n=2300)	Yes (n=677)
Screen time ≥3h, n (%)						
Yes	13579 (43.75)	2294 (35.97)	1940 (36.03)	1613 (36.13)	804 (34.99)	242 (35.36)
No	17152 (56.25)	4056 (64.03)	3421 (63.97)	2819 (63.87)	1496 (65.01)	435 (64.64)
***P***		<0.001	<0.001	<0.001	<0.001	<0.001
BMI, n (%)						
Normal	21352 (61.55)	4103 (55.84)	3429 (55.05)	2853 (55.45)	1437 (53.37)	413 (52.05)
Overweight/ Obesity	9379 (38.45)	2247 (44.16)	1932 (44.95)	1579 (44.55)	863 (46.63)	264 (47.95)
***P***		<0.001	<0.001	<0.001	<0.001	<0.001
Described themselves as slightly or very overweight, n (%)						
No	20731 (61.65)	3580 (49.81)	2970 (48.70)	2468 (49.15)	1240 (47.74)	352 (46.10)
Yes	10000 (38.35)	2770 (50.19)	2391 (51.30)	1964 (50.85)	1060 (52.26)	325 (53.90)
***P***		<0.001	<0.001	<0.001	<0.001	<0.001
Age, n (%)						
≤16 years	10524 (32.31)	2174 (32.35)	1852 (32.65)	1533 (32.86)	869 (35.88)	245 (34.10)
>16 years	20207 (67.69)	4176 (67.65)	3509 (67.35)	2899 (67.14)	1431 (64.12)	432 (65.90)
***P***		0.943	0.564	0.415	<0.001	0.308
Gender, n (%)						
Female	15913 (47.49)	4170 (61.92)	3608 (63.66)	2961 (63.07)	1653 (68.36)	505 (71.27)
Male	14818 (52.51)	2180 (38.08)	1753 (36.34)	1471 (36.93)	647 (31.64)	172 (28.73)
***P***		<0.001	<0.001	<0.001	<0.001	<0.001
Are you Hispanic or Latino, n (%)						
Yes	8393 (27.08)	1742 (27.15)	1442 (26.55)	1258 (28.11)	708 (30.24)	208 (30.48)
No	22338 (72.92)	4608 (72.85)	3919 (73.45)	3174 (71.89)	1592 (69.76)	469 (69.52)
***P***		0.905	0.409	0.143	<0.001	0.051
Smoking, n (%)						
Yes	3029 (10.43)	1140 (18.59)	992 (19.01)	861 (20.13)	567 (25.28)	229 (34.14)
No	27702 (89.57)	5210 (81.41)	4369 (80.99)	3571 (79.87)	1733 (74.72)	448 (65.86)
***P***		<0.001	<0.001	<0.001	<0.001	<0.001
Drinking, n (%)						
Yes	9926 (32.83)	2818 (44.58)	2436 (45.59)	2028 (45.86)	1201 (52.19)	414 (60.48)
No	20805 (67.17)	3532 (55.42)	2925 (54.41)	2404 (54.14)	1099 (47.81)	263 (39.52)
***P***		<0.001	<0.001	<0.001	<0.001	<0.001
Physical activity, n (%)						
Yes	15179 (49.14)	2993 (46.77)	2502 (46.31)	2103 (47.15)	1113 (47.95)	315 (45.21)
No	15552 (50.86)	3357 (53.23)	2859 (53.69)	2329 (52.85)	1187 (52.05)	362 (54.79)
***P***		<0.001	<0.001	0.026	0.316	<0.001
Illegal drug, n (%)						
Yes	228 (0.78)	144 (2.37)	132 (2.57)	128 (2.99)	109 (4.87)	68 (10.20)
No	30503 (99.22)	6206 (97.63)	5229 (97.43)	4304 (97.01)	2191 (95.13)	609 (89.80)
***P***		<0.001	<0.001	<0.001	<0.001	<0.001
Cyber bullying, n (%)						
Yes	4447 (14.07)	1935 (29.97)	1729 (31.76)	1425 (31.73)	933 (39.97)	333 (48.46)
No	26284 (85.93)	4415 (70.03)	3632 (68.24)	3007 (68.27)	1367 (60.03)	344 (51.54)
***P***		<0.001	<0.001	<0.001	<0.001	<0.001
School bullying, n (%)						
Yes	5826 (18.86)	2414 (37.95)	2103 (39.21)	1755 (39.64)	1061 (46.06)	371 (54.26)
No	24905 (81.14)	3936 (62.05)	3258 (60.79)	2677 (60.36)	1239 (53.94)	306 (45.74)
***P***		<0.001	<0.001	<0.001	<0.001	<0.001
School safety concern, n (%)						
Yes	1678 (5.45)	733 (11.44)	647 (11.98)	544 (12.25)	393 (16.91)	152 (22.36)
No	29053 (94.55)	5617 (88.56)	4714 (88.02)	3888 (87.75)	1907 (83.09)	525 (77.64)
***P***		<0.001	<0.001	<0.001	<0.001	<0.001
Had early sexual intercourse, n (%)						
Yes	1171 (4.09)	425 (7.24)	362 (7.26)	331 (8.07)	230 (10.81)	108 (17.19)
No	29560 (95.91)	5925 (92.76)	4999 (92.74)	4101 (91.93)	2070 (89.19)	569 (82.81)
***P***		<0.001	<0.001	<0.001	<0.001	<0.001
Sadness or hopeless, n (%)						
Yes	9929 (31.91)	4930 (77.22)	4340 (80.54)	3543 (79.72)	1999 (86.58)	625 (92.05)
No	20802 (68.09)	1420 (22.78)	1021 (19.46)	889 (20.28)	301 (13.42)	52 (7.95)
***P***		<0.001	<0.001	<0.001	<0.001	<0.001

### The associations between screen time and overweight/obesity and self-perceived overweight and suicidality

**Table 2** presents the relationships between screen time, overweight/obesity, self-perceived overweight, and suicidality. Adolescents with screen time ≥3h were related to higher odds of overweight/obesity (OR=1.27, 95%CI: 1.19-1.38) and self-perceived overweight (OR=1.38, 95%CI: 1.30-1.48) after adjusting for sex, race, smoking, school bullying, and sadness or hopeless. Adolescents with overweight/obesity (OR=1.30, 95%CI: 1.19-1.43) and self-perceived overweight (OR=1.54, 95%CI: 1.39-1.70) were associated with higher odds of suicidality after adjusting for sex, race, smoking, drinking alcohol, illegal drug, cyber bullying, school bullying, school safety concern, early sexual intercourse, and sadness or hopeless. Moreover, adolescents with screen time ≥3h were related to higher odds of suicidality after adjusting confounders (OR=1.35, 95%CI: 1.23-1.46). When further adjusted for overweight/obesity (OR=1.32, 95%CI: 1.22-1.455) or self-perceived overweight (OR=1.31, 95%CI: 1.20-1.43), adolescents with screen time ≥3h were still associated with higher odds of suicidality. Specific suicidality, including considered suicide, made a suicide plan, and attempted suicide, also supported the above results except for injurious suicide attempt (all *P*<0.05) (**Table 2**).

**Table 2:** Association between screen time, overweight/obesity, self-perceived overweight, and suicidality.

Variables	Self-perceived overweight	Overweight/obesity	Suicidality	Considered suicide	Made a suicide plan	Attempted suicide	Injurious suicide attempt
	OR (95%CI)	OR (95%CI)	OR (95%CI)	OR (95%CI)	OR (95%CI)	OR (95%CI)	OR (95%CI)
Screen time ≥3h^①^							
No	Ref	Ref					
Yes	1.38 (1.30-1.48)	1.27 (1.19-1.38)					
Screen time ≥3h^②^							
No			Ref	Ref	Ref	Ref	Ref
Yes			1.35 (1.23-1.46)	1.30 (1.19-1.42)	1.23 (1.13-1.35)	1.27 (1.12-1.45)	1.22 (0.96-1.54)
Screen time ≥3h^③^	Adjusted	–					
No			Ref	Ref	Ref	Ref	Ref
Yes			1.31 (1.20-1.43)	1.27 (1.15-1.39)	1.21 (1.11-1.32)	1.25 (1.08-1.42)	1.19 (0.94-1.51)
Screen time ≥3h^④^	–	Adjusted					
No			Ref	Ref	Ref	Ref	Ref
Yes			1.32 (1.22-1.45)	1.28 (1.16-1.40)	1.22 (1.12-1.34)	1.26 (1.11-1.43)	1.20 (0.95-1.51)
Self-perceived overweight^②^							
No			Ref	Ref	Ref	Ref	Ref
Yes			1.54 (1.39-1.70)	1.54 (1.40-1.70)	1.45 (1.30-1.63)	1.43 (1.27-1.62)	1.42 (1.16-1.73)
Overweight/obesity^②^							
No			Ref	Ref	Ref	Ref	Ref
Yes			1.30 (1.19-1.43)	1.32 (1.21-1.45)	1.27 (1.14-1.40)	1.22 (1.07-1.39)	1.30 (1.04-1.62)

OR, odds ratio; CI, confidence interval; Ref, reference. “①” adjusted for sex, race, smoking, school bullying, and sadness or hopeless; “②” adjusted for sex, race, smoking, drinking alcohol, illegal drug, cyber bullying, school bullying, school safety concern, early sexual intercourse, and sadness or hopeless; “③” further adjusted for self-perceived overweight based on “②”; “④” further adjusted for overweight/obesity based on “②”.

### Mediating effects of overweight/obesity and self-perceived overweight on the association between screen time and suicidality

**Figure 2:**
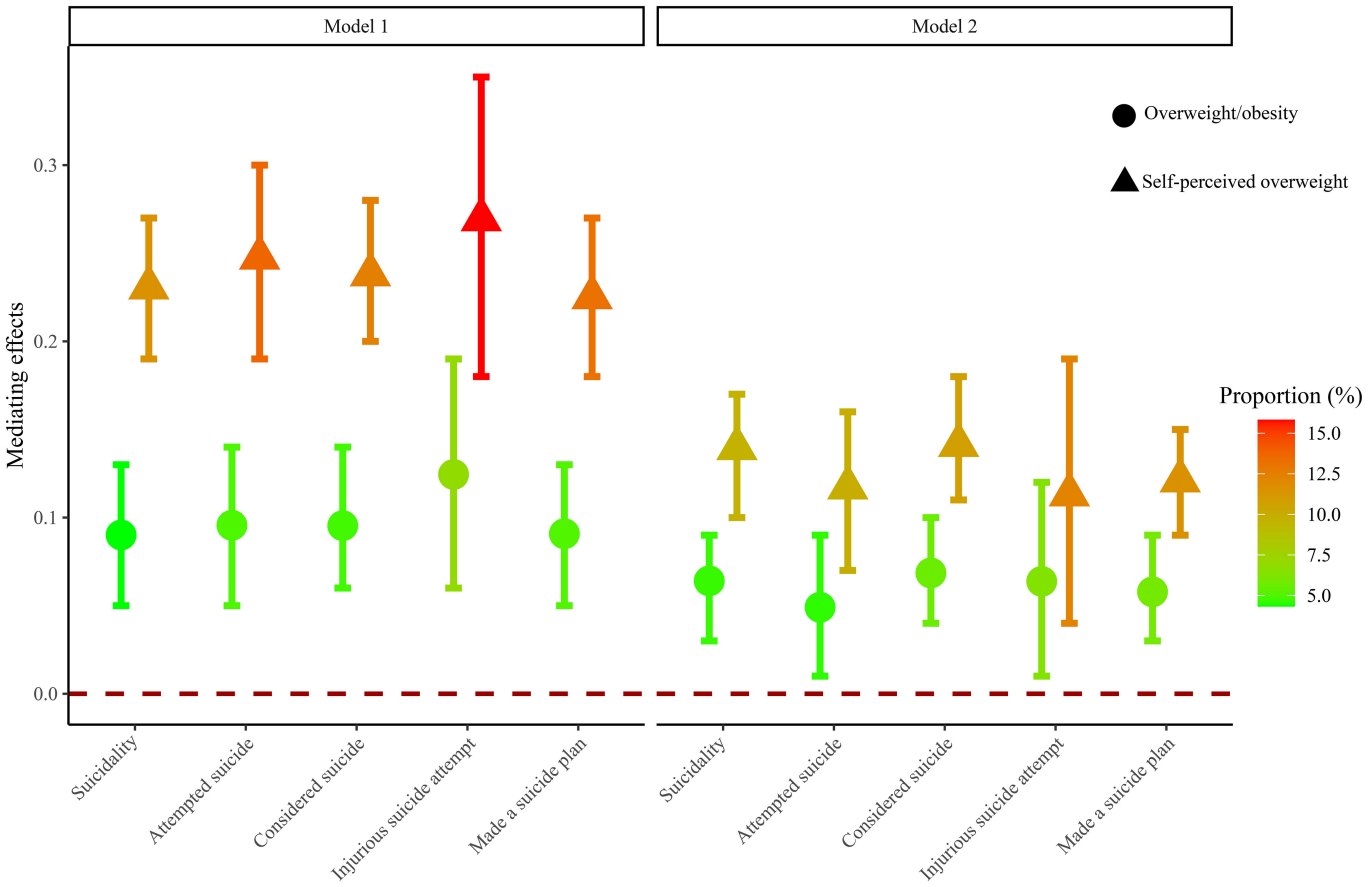
Mediating effects of overweight/obesity and self-perceived overweight on the association between screen time and suicidality. The vertical axis is the mediating effect size, and the interval without 0 indicates mediating effect. The closer the color is to red, the stronger the mediating effect. Model 1, univariable logistic regression model; Model 2, multivariable logistic regression model.

**Table 3** and **Figure 2** report the mediating effects of overweight/obesity and self-perceived overweight on the association between screen time and suicidality. In the analysis of the mediating effects between screen time, overweight/obesity, and suicidality, the direct effect was 0.28 (0.20, 0.37), the mediating effect was 0.06 (0.03, 0.09), and 4.67% of the association between screen time and suicidality was mediated by overweight/obesity. In the specific analysis of suicidality, the mediating effect of overweight/obesity was 0.07 (0.04, 0.10) and mediated 5.61% of the association between screen time and considered suicide, 0.06 (0.03, 0.09) and mediated 5.87% of the association between screen time and made a suicide plan, and 0.05 (0.01, 0.09) and mediated 4.57% of the association between screen time and attempted suicide, while no statistically significant association was found between screen time and injurious suicide attempt. In the analysis of the mediating effects between screen time, self-perceived overweight, and suicidality, the direct effect was 0.27 (0.18, 0.36), the mediating effect was 0.14 (0.10, 0.17), and 9.66% of the association between screen time and suicidality was mediated by self-perceived overweight. In the specific analysis of suicidality, the mediating effect of self-perceived overweight was 0.14 (0.11, 0.18) and mediated 10.79% of the association between screen time and considered suicide, 0.12 (0.09, 0.15) and mediated 11.57% of the association between screen time and made a suicide plan, and 0.12 (0.07, 0.16) and mediated 9.96% the association between screen time and attempted suicide. No statistically significant association was found between screen time and injurious suicide attempt (*P*>0.05).

**Table 3:** Mediating effects of overweight/obesity and self-perceived overweight on the association between screen time and suicidality.

Outcomes	Mediating variable	Effect A	Effect B	Effect C	Effect C’	Mediating effects (95% CI)	Proportion (%)
Overweight/obesity							
Considered suicide	Model 1	0.27 (0.20, 0.34)	0.35 (0.27, 0.44)	0.45 (0.37, 0.54)	0.43 (0.35, 0.52)	0.10 (0.06, 0.14)	4.8
	Model 2	0.24 (0.17, 0.32)	0.28 (0.19, 0.37)	0.26 (0.17, 0.35)	0.25 (0.15, 0.34)	0.07 (0.04, 0.1)	5.61
Made a suicide plan	Model 1	0.27 (0.20, 0.34)	0.34 (0.24, 0.43)	0.41 (0.32, 0.51)	0.39 (0.30, 0.49)	0.09 (0.05, 0.13)	5.05
	Model 2	0.24 (0.17, 0.32)	0.24 (0.13, 0.34)	0.21 (0.12, 0.30)	0.20 (0.11, 0.29)	0.06 (0.03, 0.09)	5.87
Attempted suicide	Model 1	0.27 (0.20, 0.34)	0.35 (0.24, 0.46)	0.46 (0.33, 0.59)	0.45 (0.32, 0.58)	0.10 (0.05, 0.14)	4.95
	Model 2	0.24 (0.17, 0.32)	0.20 (0.07, 0.33)	0.24 (0.11, 0.37)	0.23 (0.10, 0.36)	0.05 (0.01, 0.09)	4.57
Injurious suicide attempt	Model 1	0.27 (0.20, 0.34)	0.46 (0.25, 0.66)	0.46 (0.24, 0.68)	0.43 (0.21, 0.65)	0.12 (0.06, 0.19)	6.92
	Model 2	0.24 (0.17, 0.32)	0.26 (0.04, 0.48)	0.20 (-0.04, 0.43)	0.18 (-0.05, 0.41)	0.06 (0.01, 0.12)	6.38
Suicidality	Model 1	0.27 (0.20, 0.34)	0.33 (0.25, 0.42)	0.47 (0.38, 0.55)	0.45 (0.37, 0.53)	0.09 (0.05, 0.13)	4.33
	Model 2	0.24 (0.17, 0.32)	0.26 (0.17, 0.36)	0.30 (0.21, 0.38)	0.28 (0.20, 0.37)	0.06 (0.03, 0.09)	4.67
Self-perceived overweight							
Considered suicide	Model 1	0.36 (0.30, 0.42)	0.66 (0.59, 0.74)	0.45 (0.37, 0.54)	0.41 (0.32, 0.49)	0.24 (0.2, 0.28)	12.5
	Model 2	0.32 (0.26, 0.39)	0.43 (0.34, 0.53)	0.26 (0.17, 0.35)	0.24 (0.14, 0.33)	0.14 (0.11, 0.18)	10.79
Made a suicide plan	Model 1	0.36 (0.30, 0.42)	0.63 (0.54, 0.72)	0.41 (0.32, 0.51)	0.37 (0.27, 0.46)	0.23 (0.18, 0.27)	13.2
	Model 2	0.32 (0.26, 0.39)	0.37 (0.26, 0.49)	0.21 (0.12, 0.30)	0.19 (0.10, 0.28)	0.12 (0.09, 0.15)	11.57
Attempted suicide	Model 1	0.36 (0.30, 0.42)	0.69 (0.59, 0.79)	0.46 (0.33, 0.59)	0.41 (0.28, 0.54)	0.25 (0.19, 0.30)	13.7
	Model 2	0.32 (0.26, 0.39)	0.36 (0.24, 0.48)	0.24 (0.11, 0.37)	0.22 (0.08, 0.35)	0.12 (0.07, 0.16)	9.96
Injurious suicide attempt	Model 1	0.36 (0.30, 0.42)	0.75 (0.57, 0.94)	0.46 (0.24, 0.68)	0.40 (0.17, 0.62)	0.27 (0.18, 0.35)	15.82
	Model 2	0.32 (0.26, 0.39)	0.35 (0.15, 0.55)	0.20 (-0.04, 0.43)	0.17 (-0.06, 0.41)	0.11 (0.04, 0.19)	12.3
Suicidality	Model 1	0.36 (0.30, 0.42)	0.64 (0.57, 0.72)	0.47 (0.38, 0.55)	0.42 (0.34, 0.51)	0.23 (0.19, 0.27)	11.52
	Model 2	0.32 (0.26, 0.39)	0.43 (0.33, 0.53)	0.30 (0.21, 0.38)	0.27 (0.18, 0.36)	0.14 (0.10, 0.17)	9.66

“Effect A”, the relationship of screen time with overweight/obesity and self-perceived overweight; “Effect B”, the relationship of overweight/obesity and self-perceived overweight with suicidality; “Effect A × Effect B”, indirect effect; “Effect C” (total effect), the relationship between screen time and suicidality; “(Effect C’)” (direct effect), the relationship between screen time and suicidality after adjusting for overweight/obesity or self-perceived overweight; Standardized coefficients were utilized to show the estimates for each pathway; The proportion of mediation effect was calculated as: [exp(Effect C) - exp(Effect A)] ×100/[exp(Effect C)-1].

Model 1, univariable logistic regression model; Model 2, multivariable logistic regression model (Effect A adjusted for sex, race, smoking, school bullying, and sadness or hopeless; Effect B and Effect C adjusted for sex, race, smoking, drinking alcohol, illegal drug, cyber bullying, school bullying, school safety concern, early sexual intercourse, and sadness or hopeless; (Effect C’) further adjusted for overweight/obesity or self-perceived overweight based on Effect C).

**Figure 3:**
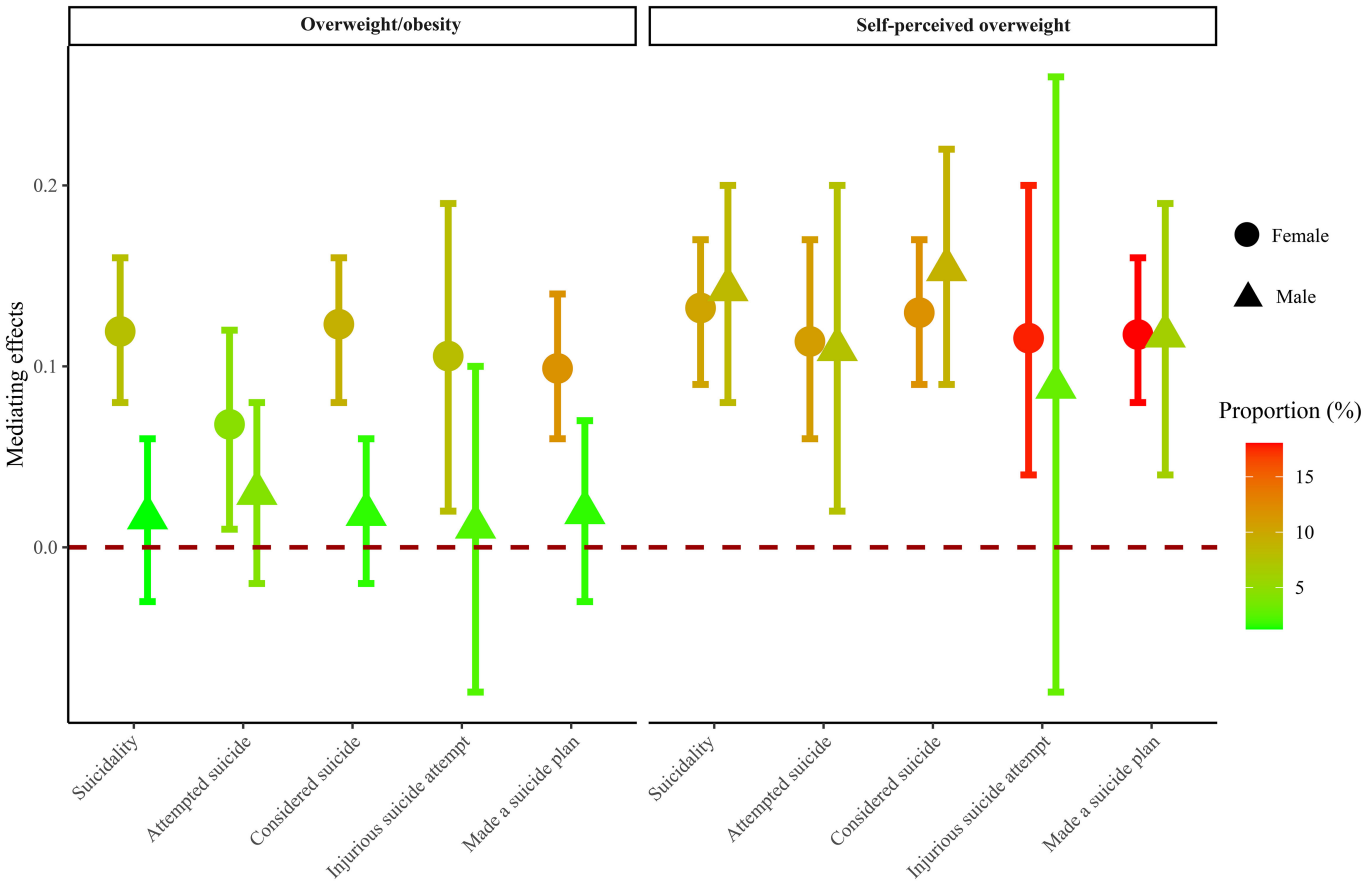
Mediating effects of overweight/obesity and self-perceived overweight based on sex. The vertical axis is the mediating effect size, and the interval without 0 indicates mediating effect. The closer the color is to red, the stronger the mediating effect. All results were multivariable logistic regression models.

**Table 4** and **Figure 3** present the mediating effects of overweight/obesity and self-perceived overweight on the association between screen time and suicidality based on sex. In the analysis of the mediating effect of overweight/obesity, 7.74% of the association between screen time and suicidality in females was mediated by overweight/obesity, whereas no mediating effect of overweight/obesity was found in males. In females, the proportion of mediating effects of overweight/obesity was 9.17%, 11.8%, and 4.52% for considered suicide, made a suicide plan, and attempted suicide, respectively. In the analysis of the mediating effect of self-perceived overweight, self-perceived overweight mediated the association between screen time and suicidality at 10.33% in females and 8.4% in males. In females, the proportion of mediating effects of self-perceived overweight was 11.87%, 18.04%, and 10.89% for considered suicide, made a suicide plan, and attempted suicide, respectively. In males, the proportion of mediating effects of self-perceived overweight was 9.00% and 6.22% for considered suicide and made a suicide plan, respectively. No association was found between screen time and injurious suicide attempt was found in females, nor between screen time and attempted suicide and injurious suicide attempt in males.

**Table 4:** Mediating effects of overweight/obesity and self-perceived overweight on the association between screen time and suicidality based on sex.

Outcomes	Mediating variable	Effect A	Effect B	Effect C	Effect C’	Mediating effects (95% CI)	Proportion (%)
Overweight/obesity							
Considered suicide	Female	0.31 (0.22, 0.41)	0.39 (0.28, 0.50)	0.23 (0.13, 0.34)	0.21 (0.11, 0.32)	0.12 (0.08, 0.16)	9.17
	Male	0.18 (0.08, 0.28)	0.10 (-0.05, 0.25)	0.32 (0.16, 0.47)	0.31 (0.16, 0.46)	0.02 (-0.02, 0.06)	1.58
Made a suicide plan	Female	0.31 (0.22, 0.41)	0.31 (0.19, 0.44)	0.14 (0.02, 0.26)	0.13 (0.00, 0.25)	0.10 (0.06, 0.14)	11.8
	Male	0.18 (0.08, 0.28)	0.11 (-0.06, 0.28)	0.34 (0.16, 0.52)	0.33 (0.15, 0.51)	0.02 (-0.03, 0.07)	1.61
Attempted suicide	Female	0.31 (0.22, 0.41)	0.22 (0.07, 0.36)	0.24 (0.09, 0.40)	0.23 (0.08, 0.39)	0.07 (0.01, 0.12)	4.52
	Male	0.18 (0.08, 0.28)	0.17 (-0.06, 0.40)	0.22 (-0.01, 0.45)	0.21 (-0.02, 0.44)	0.03 (-0.02, 0.08)	4.29
Injurious suicide attempt	Female	0.31 (0.22, 0.41)	0.34 (0.09, 0.58)	0.17 (-0.09, 0.43)	0.16 (-0.10, 0.41)	0.11 (0.02, 0.19)	8.01
	Male	0.18 (0.08, 0.28)	0.06 (-0.38, 0.50)	0.27 (-0.20, 0.75)	0.27 (-0.21, 0.74)	0.01 (-0.08, 0.1)	2.28
Suicidality	Female	0.31 (0.22, 0.41)	0.38 (0.27, 0.50)	0.28 (0.17, 0.38)	0.26 (0.15, 0.37)	0.12 (0.08, 0.16)	7.74
	Male	0.18 (0.08, 0.28)	0.09 (-0.05, 0.23)	0.33 (0.19, 0.46)	0.32 (0.18, 0.46)	0.02 (-0.03, 0.06)	1.24
Self-perceived overweight							
Considered suicide	Female	0.30 (0.21, 0.38)	0.43 (0.33, 0.54)	0.23 (0.13, 0.34)	0.21 (0.10, 0.32)	0.13 (0.09, 0.17)	11.87
	Male	0.35 (0.25, 0.45)	0.44 (0.26, 0.62)	0.32 (0.16, 0.47)	0.29 (0.14, 0.44)	0.15 (0.09, 0.22)	9
Made a suicide plan	Female	0.30 (0.21, 0.38)	0.39 (0.26, 0.52)	0.14 (0.02, 0.26)	0.12 (-0.01, 0.24)	0.12 (0.08, 0.16)	18.04
	Male	0.35 (0.25, 0.45)	0.33 (0.15, 0.52)	0.34 (0.16, 0.52)	0.32 (0.14, 0.50)	0.12 (0.04, 0.19)	6.22
Attempted suicide	Female	0.30 (0.21, 0.38)	0.38 (0.24, 0.52)	0.24 (0.09, 0.40)	0.22 (0.06, 0.38)	0.11 (0.06, 0.17)	10.89
	Male	0.35 (0.25, 0.45)	0.31 (0.04, 0.59)	0.22 (-0.01, 0.45)	0.20 (-0.03, 0.44)	0.11 (0.02, 0.2)	7.59
Injurious suicide attempt	Female	0.30 (0.21, 0.38)	0.39 (0.13, 0.64)	0.17 (-0.09, 0.43)	0.14 (-0.12, 0.40)	0.12 (0.04, 0.2)	17.68
	Male	0.35 (0.25, 0.45)	0.25 (-0.20, 0.70)	0.27 (-0.20, 0.75)	0.26 (-0.21, 0.74)	0.09 (-0.08, 0.26)	2.96
Suicidality	Female	0.30 (0.21, 0.38)	0.44 (0.33, 0.55)	0.28 (0.17, 0.38)	0.25 (0.14, 0.36)	0.13 (0.09, 0.17)	10.33
	Male	0.35 (0.25, 0.45)	0.40 (0.24, 0.57)	0.33 (0.19, 0.46)	0.30 (0.16, 0.44)	0.14 (0.08, 0.2)	8.4

“Effect A”, the relationship of screen time with overweight/obesity and self-perceived overweight; “Effect B”, the relationship of overweight/obesity and self-perceived overweight with suicidality; “Effect A × Effect B”, indirect effect; “Effect C” (total effect), the relationship between screen time and suicidality; “(Effect C’)” (direct effect), the relationship between screen time and suicidality after adjusting for overweight/obesity or self-perceived overweight; Standardized coefficients were utilized to show the estimates for each pathway; The proportion of mediation effect was calculated as: [exp(Effect C) - exp(Effect A)] ×100/[exp(Effect C)-1].

All results were multivariable logistic regression models (Effect A adjusted for sex, race, smoking, school bullying, and sadness or hopeless; Effect B and Effect C adjusted for sex, race, smoking, drinking alcohol, illegal drug, cyber bullying, school bullying, school safety concern, early sexual intercourse, and sadness or hopeless; (Effect C’) further adjusted for overweight/obesity or self-perceived overweight based on Effect C).

## Discussion

This study used the survey data of YRBSS from 2013 to 2019 to explore the association between screen time and overweight/obesity and self-perceived overweight and suicidality. The results found that screen time and overweight/obesity and self-perceived overweight were related to higher odds of suicidality. Overweight/obesity and self-perceived overweight may mediate the association between screen time and suicidality, with 4.67% of the association mediated by overweight/obesity and 9.66% by self-perceived overweight. Overweight/obesity mediated 5.61%, 5.87%, and 4.57% of the associations between screen time and considered suicide, made a suicide plan, and attempted suicide. Self-perceived overweight mediated 10.79%, 11.57%, and 9.96% of the associations between screen time and considered suicide, made a suicide plan, and attempted suicide. In different sexes, the mediating effects of overweight/obesity was observed only in females, whereas the mediating effects of self-perceived overweight had no sex difference.

The association between screen time and suicidality has been reported in previous studies (24–26). Chau et al. found that excessive screen time was associated with an increased risk of school-behavior-mental-health difficulties among adolescents, including suicide attempt (24). A 10-year longitudinal study by Coyne et al. showed that a high level of social media or television use in early adolescence was the most predictive factor for suicide risk in early adulthood (25). Liu et al. demonstrated that heavy television/videogame or non-educational computer use in a typical school day was related to a higher risk of suicidal ideation in adolescents (26). Our results suggested that adolescents with screen time ≥3h were related to higher odds of suicidality, which was consistent with previous studies. A systematic review summarized the harms of screen time for children and adolescents (16). Excessive screen time was linked to poor sleep quality, obesity, poor stress regulation (high sympathetic arousal and cortisol dysregulation), and insulin resistance (16). Moreover, excessive screen time also induces adverse psychological and psychoneurological effects such as depressive symptoms, suicidality, reduced social coping, and brain structural changes related to cognitive control and emotion regulation (27–29). These studies suggested that the influence of screen time on adolescent suicidality is multimodal, but the specific mechanism between screen time and suicidality remains unclear.

Overweight and self-perceived overweight were also reported to be associated with the risk of suicidality (6, 8, 9). Haynes et al. found that the deleterious effect of overweight on suicidality depends largely on whether a person perceives as overweight (9). The study of Daly et al. showed that self-perceived overweight is a risk factor for suicidality, independent of objective weight status (8). The current study analyzed the association between screen time and overweight/obesity and self-perceived overweight and suicidality. The results showed that both overweight/obesity and self-perceived overweight were related to higher odds of suicidality. Moreover, overweight/obesity and self-perceived overweight mediated the relationship between screen time and suicidality, with 4.67% of the association mediated by overweight/obesity and 9.66% by self-perceived overweight. The potential explanation for this finding was that screen time increased obesity risk in children and adolescents (30, 31). Adolescents who are overweight or self-perceived overweight fear negative social evaluations and stress from others, and these harmful psychological effects may trigger distress and suicidality (32, 33). In addition, our study found that the mediating role of overweight/obesity in the association between screen time and suicidality was observed only in females, whereas there were no sex differences in the mediating effect of self-perceived overweight. This may be related to women’s higher rate of suicidal behavior and more like to develop internalizing disorders such as anxiety and mood disorders (34). These internalizing disorders have been reported to potentially mediate suicidal thoughts and behaviors (35, 36). However, specific explanations require more follow-up research.

This study has several strengths. First, this study used a national sample of adolescents, which included four surveys from 2013 to 2019, and was well-represented. Then, this study explored the effects of overweight/obesity and self-perceived overweight separately, and further sex-based analyses were performed. However, several limitations of this study should be considered when interpreting the results. First, the causal relationships between screen time, overweight/obesity, self-perceived overweight, and suicidality could not be proven due to the cross-sectional study design. Second, the analyzed data were based on participants’ self-reports, which may affect the credibility of the results. Third, although this study fully accounted for the existing confounders in the database, there are still some potential confounders that may affect the results. Fourth, although this study was based on nationally representative data of US adolescents, it is uncertain whether the results can be generalized to other regions.

## Conclusions

This study analyzed the association between screen time and overweight/obesity and self-perceived overweight and suicidality among adolescents based on nationally representative data. The results supported the hypothesis that overweight/obesity and self-perceived overweight mediated the association between screen time and suicidality. Adolescents with excessive screen time and overweight or self-perceived overweight should be given greater attention to their suicide risk.

## Data availability statement

Publicly available datasets were analyzed in this study. This data can be found here: YRBSS database, https://www.cdc.gov/healthyyouth/data/yrbs/overview.htm.

## Ethics statement

The requirement of ethical approval was waived by The Second Hospital of Shanxi Medical University for the studies involving humans because the data was accessed from YRBSS (a publicly available database). The studies were conducted in accordance with the local legislation and institutional requirements. Written informed consent for participation in this study was provided by the participants’ legal guardians/next of kin.

## Author contributions

HG: Conceptualization, Project administration, Supervision, Writing – original draft, Writing – review & editing. YW: Data curation, Formal analysis, Investigation, Methodology, Writing – review & editing. XW: Data curation, Formal analysis, Investigation, Methodology, Writing – review & editing. MG: Data curation, Formal analysis, Investigation, Methodology, Writing – review & editing.
